# Long-term remission of AIDS-related primary central nervous system lymphoma in a patient under antiretroviral therapy: a case report and review of the literature

**DOI:** 10.1186/s12981-021-00403-6

**Published:** 2021-10-19

**Authors:** Pieter-Jan Gijs, Olivier Clerc

**Affiliations:** 1 Service de Médecine Interne, Université de Lausanne et Centre Hospitalier Universitaire Vaudois, Lausanne, Switzerland; 2 Service des Maladies Infectieuses, Réseau Hospitalier Neuchâtelois, Neuchâtel, Switzerland

**Keywords:** Acquired immunodeficiency syndrome, Primary central nervous system lymphoma, Antiretroviral therapy, Whole-brain radiotherapy

## Abstract

**Background:**

AIDS-related primary central nervous system lymphoma (AR-PCNSL) is an AIDS-defining disease that usually occurs when the CD4 count is less than 50 cells/μl. The frequency of the disease has substantially decreased in the era of highly active antiretroviral therapy (HAART). Prognosis is poor with rapid progression leading to death within 2–3 months if left untreated.

**Case description:**

A 65 years old male presented to medical attention with gait disturbance, weight loss and slight left-sided hemiparesis. Human immunodeficiency virus infection was diagnosed with an initial CD4 count of 116 cells/µl and a viral load of 260,000 copies/ml. Magnetic resonance imaging of the brain revealed three brain lesions involving the right frontal lobe and the left parietal lobe, which on biopsy led to a diagnosis of AR-PCNSL. HAART was initiated with whole-brain radiotherapy (WBRT), and the patient declined systemic chemotherapy. Due to poor performance status, he was transferred to palliative care. Under HAART, he slowly recovered with normalization of CD4 count and undetectable viral load. Medical imaging showed complete remission (CR) of the brain lesions. At 3-year follow-up, the patient remains in CR, but presented mild neurocognitive dysfunction possibly secondary to WBRT.

**Conclusion:**

Nowadays, treatment paradigm parallels that of primary central nervous system lymphoma in the immunocompetent population based on systemic chemotherapy (primarily high-dose intravenous methotrexate and steroids) in association with HAART. The role of WBRT is questionable because of late neurotoxic effects.

## Background

Primary central nervous system lymphoma (PCNSL) is a rare variant of extra-nodal non-Hodgkin’s lymphoma. It is an acquired immune deficiency syndrome (AIDS)-defining disease since 1983 [1] and the majority of cases in human immunodeficiency virus (HIV) patients are Epstein-Barr virus (EBV)-related [2]. AIDS-related PCNSL (AR-PCNSL) generally occurs late in the natural history of HIV infection and is usually associated with a CD4 cell count less than 50 cells/µl [3, 4] with a mean CD4 cell count of 14 cells/µl, but cases above 50 cells/µl have also been described [5]. Historically, prognosis of AR-PCNSL was poor and median survival rarely exceeded 3 months [6, 7].

The AIDS epidemic during the early 1990s led to a high incidence of PCNSL with 47.2% of CNS lymphomas occurring in AIDS-patients [8]. After the introduction of highly active antiretroviral therapy (HAART) in 1996, a decrease in incidence of AIDS was observed, including in AIDS-defining cancers. Notably, incidence of PCNSL lymphomas declined from 1.7 cases per 1000 per year in the pre-HAART era to 0.7 cases per 1000 per year in the HAART era [9]. This is probably related to better control of oncogenic viruses like EBV [10].

Presently, treatment paradigm of AR-PCNSL for patients under HAART tends to become similar to that of the immunocompetent population and includes high-dose methotrexate (HD-MTX)-based chemotherapy combined or not with whole-brain radiotherapy (WBRT) [11, 12]. The addition of HAART with conventional therapy was associated with increased survival in several retrospective studies [13–15]. Numerous anecdotal reports also showed complete remission (CR) with HAART therapy alone [16, 17].

Herein, we report a patient with an AR-PCNSL who experienced a CR on treatment with HAART and WBRT, after refusal of systemic chemotherapy. We then reviewed other cases of AR-PCNSL to assess modalities of treatment associated with CR.

## Case presentation

**Fig. 1:**
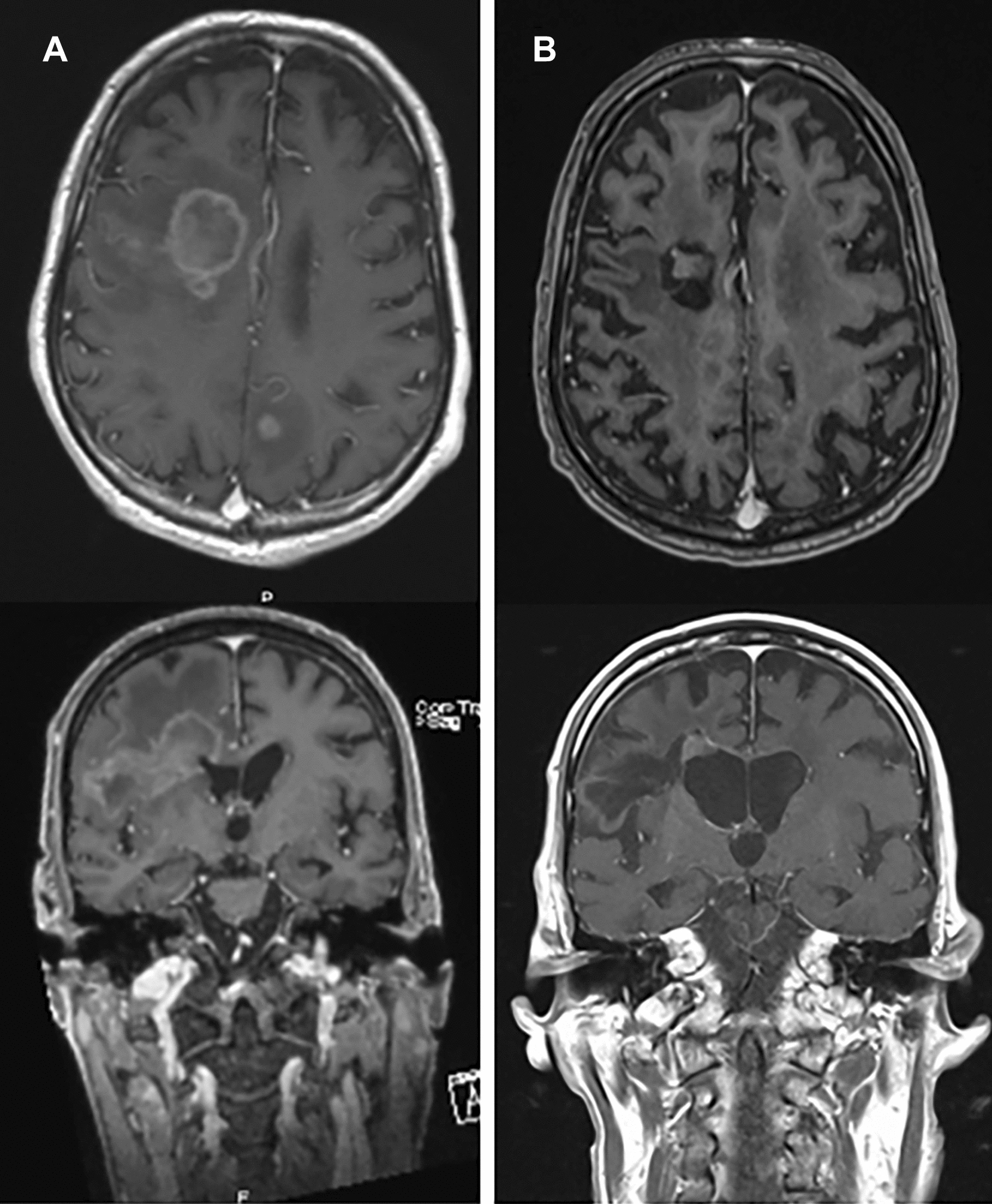
**A** T1-weighted with Gadolinium axial and coronal magnetic resonance image of brain showing a ring enhanced brain lesion involving the right frontal lobe. **B** T1-weighted with Gadolinium axial and coronal magnetic resonance image showing a complete remission of the lesion

A 65 years-old Caucasian man, without significant medical history, was admitted to the hospital in May 2017 for progressive gait disturbance, diffuse paresthesias of the left arm and a weight loss of 30 kg over the past year. Neurologic examination revealed a slight left hemiparesis at the outstretched arm and Mingazzini tests without specific motor or sensory deficit. HIV screening test returned positive with CD4 cell count of 116 cells/µl and viral load of 260,000 copies/ml. Brain magnetic resonance imaging (MRI) showed two lesions involving the right frontal lobe with slight mass effect on the right ventricle and a third lesion in the left parietal lobe (Fig. 1a).

Differential diagnosis of focal lesions in a severely immunosuppressed patient included mainly cerebral toxoplasmosis and PCNSL. As toxoplasmosis serology was positive, empiric treatment for toxoplasmosis (pyrimethamine and sulfadiazine) was initiated. Under treatment, left hemiparesis worsened and the patient developed dysarthria. A stereotactic biopsy was then performed and histopathologic examination revealed diffuse large B-cell lymphoma positive for EBV leading to a diagnosis of AR-PCNSL.

At first, systemic chemotherapy (methotrexate, cytarabine, thiotepa and rituximab) was started but had to be stopped because of patient's refusal to pursue therapy and due to poor performance status after one cycle. Antiretroviral treatment including abacavir, lamivudine and dolutegravir was started and the patient received WBRT (30 Grays in total). The patient refused any other oncological treatment and, due to poor performance status and estimated very low life expectancy, he was transferred to palliative care.

However, the patient slowly recovered with progressive resolution of the left-sided hemiparesis. Follow-up brain MRIs showed progressive improvement, and ultimately CR of the brain lesions (Fig. 1b). Regression of brain lesions was associated with immune restoration under HAART with CD4 cell count rapidly increasing above 200 cells/µl (417 cells/µl at CR). Concomitantly, patient performance status normalized and the patient was able to leave the hospital and followed in outpatient care.

Three years after initial presentation, the patient was still in CR under HAART, with undetectable viral load and a CD4 count of 364 cells/µl. Notably, he now presents with mild neurocognitive dysfunction possibly secondary to WBRT.

## Discussion

Due to the rarity of the disease, prospective studies regarding treatment of AR-PCNSL are very scarce and literature consists mainly of retrospective studies and case series. In immunocompetent hosts, treatment of PCNSL has evolved from radiotherapy alone to HD-MTX-based chemotherapy with or without radiotherapy [18]. Indeed, WBRT has been associated with irreversible cognitive dysfunction [19, 20]. Patients commonly present at diagnosis with a poor functional status, which impairs chemotherapy planning, as was the case for our patient.

Most studies regarding chemotherapy in AR-PCNSL involved the pre-HAART era and were retrospectively analyzed. Several studies showed that HD-MTX alone or combined with other agents was active and relatively well tolerated with a response rate between 30 to 57% but a low overall survival of about 3 months [21, 22].

More recently, Gupta et al. described in the HAART era a multi-center retrospective cohort of 20 patients with AR-PCNSL treated with HD-MTX (alone or in combination with other agents) and HAART without WBRT, with an overall survival and progression free survival that exceeded 60 months [23]. Chemotherapy was relatively well tolerated except for 2 deaths secondary to sepsis during induction in patients with poor performance status. Moulignier et al. confirmed these findings showing that combined HD-MTX monotherapy and optimal HAART effectively treated AR-PCNSL with a median overall survival of 5.7 years [24]. These result compared favorably with the pre-HAART era.

Other immunochemotherapeutic approaches that combined HAART, HD-MTX and rituximab without WBRT also led to long-term remission in more than 70% of patients [25].

WBRT alone has been for a long time the gold standard treatment of AR-PCNSL. Several studies in the pre-HAART era showed that WBRT was effective, especially with doses ≥ 30 Grays, but responses were generally short-lived. Indeed, median survival after radiotherapy was reported between 2 and 5 months [11, 26, 27]. In the HAART era, several studies showed improved overall survival in AR-PCNSL for patients treated with WBRT and HAART [13, 14, 28]. A recent retrospective cohort study of 23 patients with AR-PCNSL between 2002 and 2008 treated with HAART and WBRT showed a 3-year overall survival of 64% and that WBRT had an independent positive impact on survival. Performance status was also a major independent factor of survival as patients with good performance status had a 3-year overall surviving rate of 100% against only 38% in patients with poor performance status [28]. Importantly, 21% of patients that survived more than 12 months after radiation developed side effects such as leukoencephalopathy (grade ≥ 2). This is a real concern, as patients with AR-PCNSL might be particularly susceptible for radiation-induced brain injury [14] especially when neurocognitive disorder is already a well-known CNS complication of HIV disease [29].

Finally, HAART is now an important part of treatment of AR-PCSNL. The majority of AR-PCSNL are EBV related and it is accompanied by impaired specific T-cell responses against EBV antigens [30]. Treatment of HIV infection leading to immune restoration allows better immunoregulation of EBV infection and thus, potentially, reversal of immune impairment may contribute to the treatment of brain lymphoma. Similarly, HAART with good CNS penetration may better protect the brain from HIV-related injury and cognitive impairment by reducing cerebro-spinal fluid viral load [29]. This supports the hypothesis of a potential effect of HAART on EBV-infected cells in AR-PCNSL.

McGowan and Shah published the first case report that showed CR under HAART alone [31]. This report was followed with several other reports showing the same results [3, 16, 32]. Recently, Alvarez-Pinzon et al. also showed CR with combined HAART and Gamma knife radiosurgery [33]. Several multicenter retrospective series also showed improvement in survival in patients treated with HAART. Hoffman et al. studied 29 HIV-infected patients with histologically confirmed PCNSL. In this cohort, 12 of the 29 patients were treated with WBRT alone, 6 with HAART alone and 11 did not receive any treatment. Survival of patients treated with HAART differed significantly from those receiving no therapy (1093 and 33 days, respectively) [14]. These findings were confirmed in two other retrospectives series [13, 34].

## Conclusion

In summary, optimal therapeutic approach for AR-PCNSL remains unclear due to the rarity of the disease and the paucity of prospective studies to guide clinical management. WBRT long stood as the gold standard therapy but now its potential long-term consequences on neurologic and cognitive function are important features to consider.

Nowadays, treatment modalities are based on systemic chemotherapies that parallel the treatment of PCNSL in the general population, in association with HAART. Several reports showed successful treatment with HAART alone and that it can provide long-term survival even in patients with advanced HIV disease. Given that, complementary treatment should be guided by performance status, and the short-term risk of progression and death for patients with AR-PCNSL should be balanced against the risk of long-term complications of other treatments modalities. Our case report suggests WBRT might impact long-term neurological recovery and its systematic use should be questioned in the current era of HAART.

## Data Availability

Not applicable.

## Abbreviations

PCNSL: Primary central nervous system lymphoma
AIDS: Acquired immunodeficiency syndrome
AR-PCNSL: AIDS-related primary central nervous system lymphoma
HAART: Highly active antiretroviral therapy
WBRT: Whole-brain radiotherapy
EBV: Epstein-Barr virus
HIV: Human immunodeficiency virus
MRI: Magnetic resonance imaging
CR: Complete remission

## References

[1] Castro K, Ward J, Slutsker L, Buehler J, Jaffe H, Berkelman R (1993). 1993 Revised classification system for HIV infection and expanded surveillance case definition for AIDS among adolescents and adults: centers for disease control and prevention. Lab Med.

[2] Bashir R, McManus B, Cunningham C, Weisenburger D, Hochberg F (1994). Detection of Eber-1 RNA in primary brain lymphomas in immunocompetent and immunocompromised patients. J Neurooncol.

[3] Kasamon YL, Ambinder RF (2005). AIDS-related primary central nervous system lymphoma. Hematol Oncol Clin North Am.

[4] Singer EJ, Valdes-Sueiras M, Commins D, Levine A (2010). Neurologic presentations of AIDS. Neurol Clin.

[5] Gopal S, Patel MR, Yanik EL, Cole SR, Achenbach CJ, Napravnik S (2013). Temporal trends in presentation and survival for HIV-associated lymphoma in the antiretroviral therapy era. J Natl Cancer Inst.

[6] Forsyth PA, DeAngelis LM (1996). Biology and management of AIDS-associated primary CNS lymphomas. Hematol Oncol Clin North Am.

[7] Raez LE, Patel P, Feun L, Restrepo A, Raub WA, Cassileth PA (1998). Natural history and prognostic factors for survival in patients with acquired immune deficiency syndrome (AIDS)-related primary central nervous system lymphoma (PCNSL). Crit Rev Oncog.

[8] Shiels MS, Pfeiffer RM, Hall HI, Li J, Goedert JJ, Morton LM (2011). Proportions of kaposi sarcoma, selected non-hodgkin lymphomas, and cervical cancer in the United States occurring in persons with AIDS, 1980–2007. JAMA - J Am Med Assoc.

[9] International Collaboration on HIV and Cancer (2000). Highly active antiretroviral therapy and incidence of cancer in human immunodeficiency virus-infected adults. J Natl Cancer Inst.

[10] Bellan C, De Falco G, Lazzi S, Leoncini L (2003). Pathologic aspects of AIDS malignancies. Oncogene.

[11] González-Aguilar A, Soto-Hernández JL (2011). The management of primary central nervous system lymphoma related to AIDS in the HAART era. Curr Opin Oncol.

[12] Yarchoan R, Uldrick TS (2018). HIV-associated cancers and related diseases. N Engl J Med.

[13] Skiest DJ, Crosby C (2003). Survival is prolonged by highly active antiretroviral therapy in AIDS patients with primary central nervous system lymphoma. AIDS.

[14] Hoffmann C, Tabrizian S, Wolf E, Eggers C, Stoehr A, Plettenberg A (2001). Survival of AIDS patients with primary central nervous system lymphoma is dramatically improved by HAART-induced immune recovery. AIDS.

[15] Uldrick TS, Pipkin S, Scheer S, Hessol NA (2014). Factors associated with survival among patients with AIDS-related primary central nervous system lymphoma. AIDS.

[16] Aboulafia DM, Puswella AL (2007). Case report: highly active antiretroviral therapy as the sole treatment for AIDS-related primary central nervous system lymphoma: a case report with implications for treatment. AIDS Patient Care STDS.

[17] Travi G, Ferreri AJM, Cinque P, Gerevini S, Ponzoni M, Terreni MR (2012). Long-term remission of HIV-associated primary CNS lymphoma achieved with highly active antiretroviral therapy alone. J Clin Oncol.

[18] Brandsma D, Bromberg JEC (2018). Primary CNS lymphoma in HIV infection. Handb Clin Neurol..

[19] Sun A, Bae K, Gore EM, Movsas B, Wong SJ, Meyers CA (2011). Phase III trial of prophylactic cranial irradiation compared with observation in patients with locally advanced non-small-cell lung cancer: neurocognitive and quality-of-life analysis. J Clin Oncol.

[20] Thiel E, Korfel A, Martus P, Kanz L, Griesinger F, Rauch M (2010). High-dose methotrexate with or without whole brain radiotherapy for primary CNS lymphoma (G-PCNSL-SG-1): a phase 3, randomised, non-inferiority trial. Lancet Oncol.

[21] Forsyth PA, Yahalom J, DeAngelis LM (1994). Combined-modality therapy in the treatment of primary central nervous system lymphoma in AIDS. Neurology.

[22] Jacomet C, Girard PM, Lebrette MG, Farese VL, Monfort L, Rozenbaum W (1997). Intravenous methotrexate for primary central nervous system non-Hodgkin’s lymphoma in AIDS. AIDS.

[23] Gupta NK, Nolan A, Omuro A, Reid EG, Wang CC, Mannis G (2017). Long-term survival in AIDS-related primary central nervous system lymphoma. Neuro Oncol.

[24] Moulignier A, Lamirel C, Picard H, Lebrette M-G, Amiel C, Hamidi M (2017). Long-term AIDS-related PCNSL outcomes with HD-MTX and combined antiretroviral therapy. Neurology.

[25] Lurain K, Uldrick TS, Goncalves PH, Ramaswami R, Polizzotto MN, Widell A (2018). Radiation-sparing treatment of HIV-related primary central nervous system lymphoma with antiretroviral therapy, rituximab and high-dose methotrexate. Blood.

[26] Baumgartner JE, Rachlin JR, Beckstead JH, Meeker TC, Levy RM, Wara WM (1990). Primary central nervous system lymphomas: natural history and response to radiation therapy in 55 patients with acquired immunodeficiency syndrome. J Neurosurg.

[27] Formenti SC, Gill PS, Lean E, Rarick M, Meyer PR, Boswell W (1989). Primary central nervous system lymphoma in AIDS. Results of radiation therapy. Cancer.

[28] Nagai H, Odawara T, Ajisawa A, Hagiwara S, Watanabe T, Uehira T (2010). Whole brain radiation alone produces favourable outcomes for AIDS-related primary central nervous system lymphoma in the HAART era. Eur J Haematol.

[29] Letendre S (2011). Central nervous system complications in HIV disease: HIV-associated neurocognitive disorder. Top Antivir Med.

[30] Gasser O, Bihl FK, Wolbers M, Loggi E, Steffen I, Hirsch HH (2007). HIV patients developing primary CNS lymphoma lack EBV-specific CD4 + T cell function irrespective of absolute CD4+ T cell counts. PLoS Med..

[31] McGowan JP, Shah S (1998). Long-term remission of AIDS-related primary central nervous system lymphoma associated with highly active antiretroviral therapy. AIDS.

[32] Chotmongkol V, Pesee M (2002). AIDS-related primary central nervous system lymphoma: prolonged remission associated with highly active antiretroviral therapy. J Med Assoc Thail.

[33] Alvarez-Pinzon AM, Valerio JE, Swedberg HN, Elwasila SM, Wolf A, Alonso JR (2019). Highly active antiretroviral therapy and gamma knife radiosurgery for the treatment of AIDS-related primary central nervous system lymphoma. World Neurosurg.

[34] Newell ME, Hoy JF, Cooper SG, DeGraaff B, Grulich AE, Bryant M (2004). Human immunodeficiency virus-related primary central nervous system lymphoma: factors influencing survival in 111 patients highly active antiretroviral therapy and gamma knife radiosurgery for the treatment of AIDS-related primary central nervous system lymphoma. Cancer.

